# The burden of mental health-related mortality in the Baltic States in 2007-2018

**DOI:** 10.1186/s12889-022-14175-9

**Published:** 2022-09-19

**Authors:** Daumantas Stumbrys, Domantas Jasilionis, Dainius Pūras

**Affiliations:** 1 Institute of Sociology and Social Work, Faculty of Philosophy, Vilnius University, Universiteto Street. 9, LT-01513 Vilnius, Lithuania; 2 Demographic Data Laboratory, Max Planck Institute for Demographic Research, Rostock, Germany; 3 Demographic Research Centre, Vytautas Magnus University, Kaunas, Lithuania; 4 Faculty of Medicine, Vilnius University, Vilnius, Lithuania

**Keywords:** Alcohol, Baltic States, Estonia, Latvia, Lithuania, Mental health, Mortality, Suicide, Years of life lost

## Abstract

**Background:**

The problem of underestimating the burden of mental health-related mortality is widely discussed in the public health literature. Relevant scientific evidence from societies experiencing the largest burden of mental health mortality is important for better understanding global and national mental health challenges and improving policies. Three Baltic States - Estonia, Lithuania, and Latvia - are countries in the Central and Eastern European region that experienced post-soviet transition trauma and showed among the highest suicide and alcohol-related mortality rates in Europe. This study aimed to examine the change in the burden of mental health-related mortality in three Baltic States in the context of consistent growth in life expectancy in 2007-2018.

**Methods:**

We calculated age-standardized years of life lost due to specific mental health-related causes of death in three Baltic countries from 2007 to 2018. Four mental health-related causes of death groups were analyzed: (i) all mental and behavioural disorders; (ii) intentional self-harm; (iii) main substance use-related causes of death; and (iv) external causes of death. The number of deaths came from the WHO Mortality Database; population exposures were extracted from the Human Mortality Database.

**Results:**

We found that the proportion of age-standardized years of life lost due to mental disorders was relatively low in all three countries. It varied from 0.2% for females in Lithuania in 2009 to 2.4% for males in Estonia in 2007. However, the proportion of age-standardized years of life lost from self-harm and substance use remained high. In 2018, the proportion of age-standardized years of life lost due to self-harm was highest among males in Lithuania (4.1%) while the highest proportion due to substance use-related causes of death was among males in Estonia (7.3%).

**Conclusions:**

Our findings indicate that the burden of mental health-related mortality remained high and showed divergent temporal changes across the three countries. In the context of the Baltic States and other post-soviet countries, fractions of various external causes of death and alcohol-related causes of death should be considered in assessing the total burden of mental health-related mortality.

**Supplementary Information:**

The online version contains supplementary material available at 10.1186/s12889-022-14175-9.

## Background

Scholars have an ongoing debate on the global burden of mental illness and substance use disorders [1–4]. People across the world are facing mental disorders in both sexes and across all age groups [5]. The risk of mental disorders starts in early childhood with idiopathic intellectual disability and autism spectrum disorders and continues into older ages with depressive disorders, anxiety disorders, and schizophrenia [5]. Recent findings based on the Global Burden of Disease study show that mental and addictive disorders caused 7% of all global burden of disease as measured in disability-adjusted life-years and 19% of all years lived with disability [2]. However, some researchers argue that the share of the burden related to mental illness is underestimated. Vigo et al. [1] argue that group of causes related to mental health should be expanded by causes from these groups: psychiatric and neurological disorders, suicide, chronic pain syndromes, personality disorders, and severe mental illness. Vigo et al. [1] estimate the disease burden for mental illness to show that the global burden of mental illness accounts for 32.4% of years lived with disability (YLDs) and 13.0% of disability-adjusted life-years (DALYs), instead of the earlier estimates suggesting 21.2% of YLDs and 7.1% of DALYs [6].

**Fig. 1:**
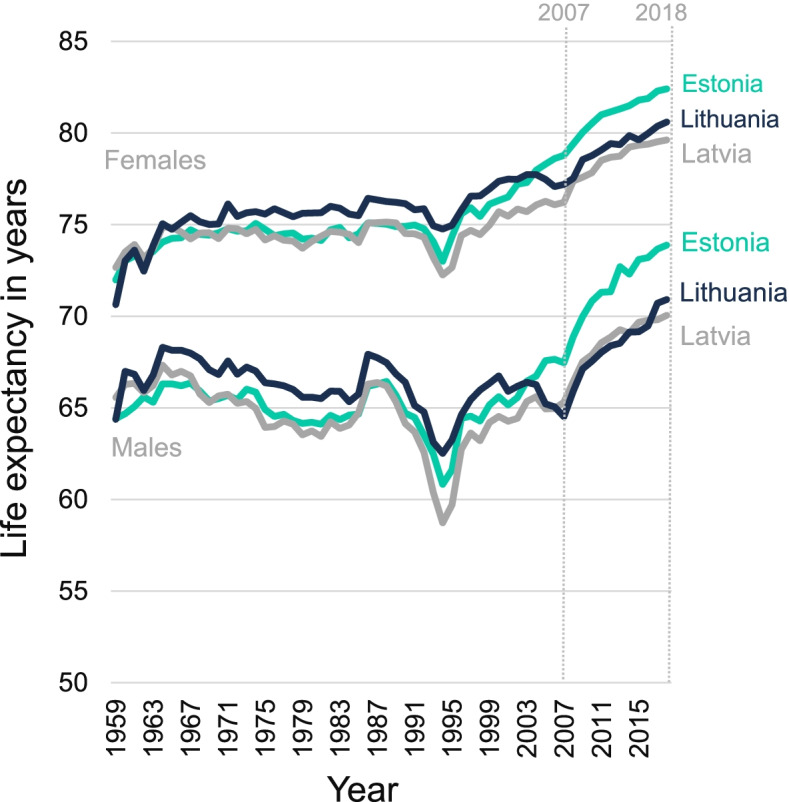
Male and female life expectancy in Estonia, Latvia, and Lithuania from 1959 to 2018. Three curves at the top of the chart represent female life expectancy changes, three curves at the bottom of the chart represent male life expectancy changes. Two vertical grey dotted lines show the start of the period in 2007 and the end of the period in 2018. Data source: the Human Mortality Database [9]

In all three Baltic States, major mental health indicators and characteristics of health care systems were quite similar in the 1990s, but they started diverging following different paths of the health system reforms [7, 8]. General mortality patterns and changes in life expectancy also started diverging in the 2000s with Estonia taking a lead in longevity improvement, whereas Latvia and especially Lithuania experienced a stagnation or even decrease in longevity [7]. However, all three Baltic States experienced consistent and remarkable growth in life expectancy from 2007 to 2018 (Fig. 1). Male life expectancy increased by 6.4 years in both Lithuania and Estonia and by 4.8 years in Latvia. Female life expectancy was growing at a similar speed during the reference period in all three countries (3.4 years for Latvia and Lithuania and 3.6 years in Estonia [9]). Although studies acknowledge the importance of mental health problems in the region, reliable population-level evidence of their impact on aggregated mortality and longevity measures is very scarce.

It is difficult to compare the public mental health status of different countries by using morbidity data or health care system indicators. Usually, it provides more information about the health care system than about the public mental health status. It has been suggested that suicide rates can be used as a surrogate indicator of the overall level of mental health [10]. Previous review has shown [11], that individuals with a mental disorder had a nearly eight-fold increased risk of suicide compared with those without a mental disorder. Moreover, Arsenault-Lapierre et al. [12] found that 87.3% (SD 10.0%, *N* = 3275) individuals who died by suicide had been diagnosed with a mental disorder prior to their death. Therefore, overal suicide mortality level is reliable and comparable public mental health indicator.

Over the past three decades, Lithuania has maintained the highest suicide rates in Europe or even globally [8]. Previous studies show that the suicide epidemic in Lithuania is associated with social suffering caused by the post-soviet transition and challenges in the implementation of an effective mental health policy [13–15]. The suicide epidemic phenomenon could be explained by the complexity of different factors and mechanisms that affected the overall mental health of the Lithuanian population [13]. For example, some studies explain poor mental health status by highlighting the role of historical traumas and consequences of social transformations [13, 14]. Furthermore, a persisting masculinity culture and traditional gender role models [16], stigmatisation of mental health problems [17], large share of the prison population and population living in big institutional social care homes may also have contributed to the unfavourable mental health situation in the Lithuanian population [15]. The mental health system has been reliant on excessive institutionalization and medicalization since the Soviet era in Lithuania [15]. After the restoration of independence in 1990, there have been attempts to modernize mental health policy and services. Lithuania has had a modern mental health strategy since 2007 [15]. However, many challenges remain in the level of implementation, promotion of human rights standards, empowerment of users of services, and independent monitoring in Lithuania.

Estonia has reached a considerable decline in suicide since 1994. The standardized suicide mortality rate per 100,000 in Estonia was the lowest (12.2) when compared to Latvia (16.3) and Lithuania (25.3) in 2016 [8]. However, suicide is one of the main causes of death among men aged 15-24 years in Estonia [18]. The Baltic States inherited the mental health care system from the Soviet Union in the 1990s. In this system, physical and mental disability was stigmatized, and most disabled people were taken into an institutionalized care setting, even when they could have lived in the community with only modest assistance [19]. In Estonia, the mental health care system has transformed into a more humane system which aims to improve patients’ quality of life in the 1990s. A new concept of social services was developed which transformed institutional care system to community care system [19].

Mental care remains focused on inpatient care in Latvia and Lithuania [20]. World Health Organization data show [8] that the number of psihciatric hospital beds per 100,000 population was exceeding the EU average in both countries. A recent analysis [20] shows that the move towards community-based mental health-care services has been slow in Latvia, and mental health-patients are still stigmatized on society. Mental health patients are often hospitalized not for medical but for social-psychological indications that could be treated at an outpatient level [20]. However, a pressure by international organizations stimulated local efforts to develop community-based mental health services. Financial resources from large psychiatric hospitals were transferred to community-based clinics recently [20].

More than one in six people across EU countries (17.3%) had a mental health problem in 2016 [21]. Across EU countries, the most common mental disorders were anxiety disorders (5.4% of the total population) depressive disorders (4.5%), drug and alcohol use disorders (2.4%), bipolar disorders (1.0%), and schizophrenic disorders (0.3%) [21]. The Baltic States were top 3 countries with the highest share of the total population reporting alcohol use disorders. The estimated prevalence of drug and alcohol use disorders was highest in Estonia (5.7%) followed by Latvia (5.0%) and Lithuania (4.8%) [21]. Previous studies have shown that alcohol and drug-related disorders are important contributors to high overall mortality rates in the Baltic States [7, 22].

Alcohol use is important factor related to high mortality from external causes of death. The Baltic States have shown extremely high mortality levels from external causes of death when compared to other EU countries [8]. Previous studies conducted in Lithuania [23] and Estonia [24] suggest that high mortality from external causes could be related to alcohol use. Alcohol in blood was found among 56.8% of individuals who died from external causes of death [23]. Moreover, it is known that mental disorders are attributed to higher risk of daying from suicide [12], homicide [25], and other external causes of death [26]. Therefore, we decided to include external causes of death in the analysis as causes related to substance use and other mental disorders.

This study aims at filling the persisting evidence gap on public health burden of mental health-related mortality in three Baltic States with the focus on the period 2007-2018. More precisely, we are exploring (i) a burden of mental health-related mortality when compared to other causes of death; (ii) temporal changes in mental health-related mortality; (iii) differences of the burden of mental health-related mortality in the context of mental health policies in the three countries.

## Methods

The period from 2007 to 2018 was selected for analysis taking into account the availability of cause-specific data in the WHO Mortality Database. Since we focus on the potential role of mental health in the systematic improvement of life expectancy in three countries, we choose the year 2007 as it was a starting point of consistent change in life expectancy after the period of fluctuations in Latvia and Lithuania. The number of deaths came from the WHO Mortality Database which is reporting mortality data annually from civil registration systems of different countries [27]. Population exposures were extracted from the Human Mortality Database [9]. In the analysis, deaths and population exposure were divided into five-year age intervals from 0 to 85+ years. Deaths with unknown ages were excluded from the analysis. The number of deaths with unknown age constituted less than 0.01% of all deaths.

We calculated age-standardized years of life lost (ASYR) due to specific mental health and substance use-related cause of death groups in three Baltic countries from 2007 to 2018. Years of life lost due to premature deaths is acknowledged as an accurate measure for assessing the impact of specific causes of death on premature mortality [28]. The calculation of the years of life lost is based on the difference between age at death and the standard life expectancy at that age [28]. We choose a standard life expectancy for the year 2050 provided by WHO Global Health Estimates [29]. It is projected that the highest national life expectancy will reach 91.94 years at birth in 2050 [29]. Furthermore, years of life lost went through standardization procedure which ensured comparability across three Baltic States with different population age structures. The standard European population of 2013 [30] was applied in calculations. To sum up, the ASYR shall be interpreted as the number of years of life lost due to a given premature mortality per population as if all three countries have the same population age distribution of the standard population [28].

In our analysis, causes of death were classified according to the International Statistical Classification of Diseases and Related Health Problems, 10th edition. Overall eight causes of death groups were analysed. Four of them were directly or underictly related to mental health: (i) all mental and behavioural disorders (F01-F99); (ii) intentional self-harm (X60-X84); (iii) main substance use related causes of death: alcoholic liver disease (K70), fibrosis and cirrhosis of the liver (K74), accidental poisoning by alcohol and drugs (X42, X45), alcoholic cardiomyopathy (I42.6), degeneration of nervous system due to alcohol (G31.2), alcoholic polyneuropathy (G62.1); (iv) all external causes of death not mentioned in previous groups. Four more groups of causes were analysed as other major causes of death groups: (v) diseases of the circulatory system (I00-I99) not mentioned in previous groups; (vi) neoplasms (C00-D48); (vii) infectious diseases (A00-B99) and diseases of the respiratory system (J00-J99); (viii) all other causes of death not mentioned in previous groups.

## Results

**Fig. 2:**
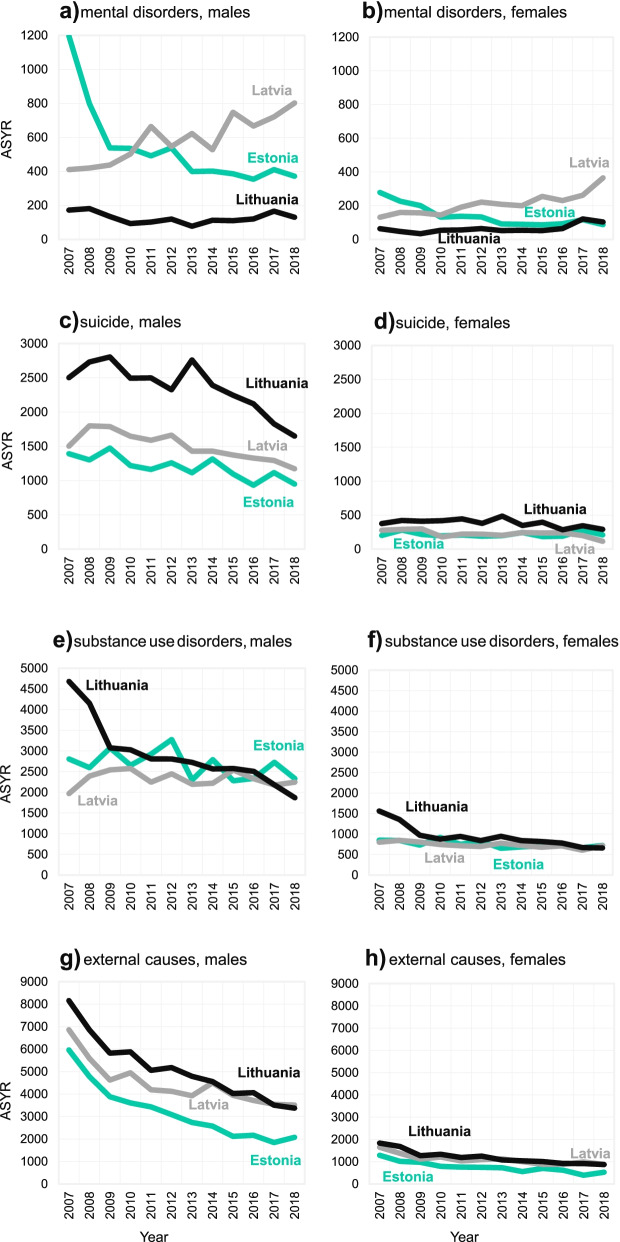
Change in age-standardized years of life lost from mental health-related causes of death groups, in Estonia, Latvia, and Lithuania in 2007-2018. Standard life expectancy for the year 2050 provided by WHO Global Health Estimates [29] and Standard European population of 2013 [30] was applied in calculations

**Fig. 3:**
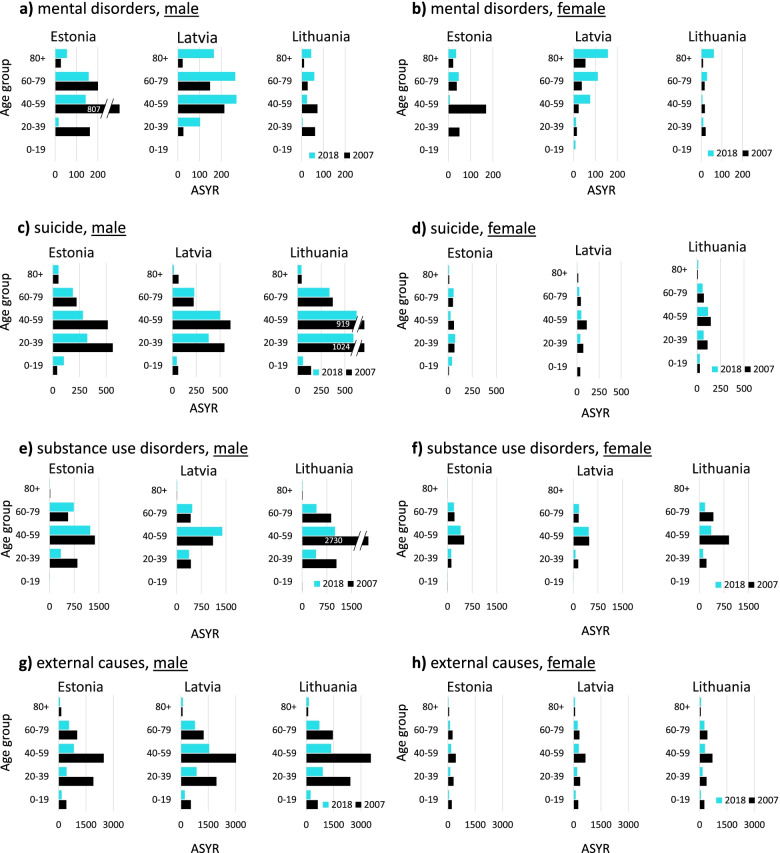
Composition of age-standardized years of life lost from mental health-related causes of death groups by age group, in Estonia, Latvia, and Lithuania in 2007 and 2018. Standard life expectancy for the year 2050 provided by WHO Global Health Estimates [29] and Standard European population of 2013 [30] was applied in calculations

Several patterns are worth mentioning from the analysis of the burden of mental health disorders in the Baltic States. Figure 2 depicts premature mortality in age-standardized years of life lost (ASYR) from specific mental health-related causes of death groups, in Estonia, Latvia, and Lithuania in 2007-2018. First, results show that the burden of mental health-related mortality in ASYR was much heavier for males when compared to females in all four causes of death groups in all Baltic countries. Second, changes in ASYR from mental disorders show country-specific patterns. ASYR changes in Estonia and Latvia went to opposite directions. Increase in Latvia versus sudden decrease and stagnation in Estonia (Fig. 2a and b). At the same time, Lithuania experienced systematic stagnation. The proportion of ASYR from mental disorders in all three countries was relatively small when compared to other causes of death. It varied from 0.2% for females in Lithuania in 2009 to 2.4% for males in Estonia in 2007 (see Table 1; for additional details on the calculation of age-standardized years of life lost, see Additional file 1). Third, causes of death structure in mental disroders group (F00-F99) varied a lot among all three countries. For example, the dominant cause of death was mental and behavioural disorders due to use of alcohol (F10) in mental disorders group (86.6%) in Estonia in 2007. While in Latvia the dominant causes of deaths (48.6%) were dementias (F01, F03) in the same period. Fourth, the burden of mental disorders-related mortality is shifting from working age population (20-39 and 40-59 years) to elderly population (60+ years) in all three countries from 2007 to 2018 (Fig. 3a and b).

**Table 1:** The age-standardized years of life lost from major causes of death groups in Estonia, Latvia, and Lithuania in 2007 and 2018

Country	Year	Cause of death group							
		Mental disorders	Suicide	Substance use	External causes	CVD	Cancer	Infections and resp.	All other
**MALES**									
**Estonia**	2007	2.4% (1197)	2.8% (1391)	5.6% (2805)	11.9% (5960)	41.9% (20893)	20.5% (10210)	5.0% (2500)	9.9% (4924)
	2018	1.2% (371)	3.0% (950)	7.3% (2329)	6.5% (2075)	37.7% (12036)	28.4% (9070)	6.0% (1922)	10.0% (3186)
**Latvia**	2007	0.7% (410)	2.6% (1503)	3.4% (1969)	11.9% (6860)	46.6% (26871)	18.2% (10521)	5.3% (3081)	11.2% (6486)
	2018	1.9% (802)	2.8% (1172)	5.4% (2246)	8.4% (3510)	42.5% (17758)	22.9% (9551)	6.1% (2543)	10.0% (4185)
**Lithuania**	2007	0.3% (172)	4.3% (2502)	8.0% (4680)	13.9% (8158)	39.4% (23136)	18.1% (10646)	7.1% (4148)	9.0% (5296)
	2018	0.3% (130)	4.1% (1649)	4.7% (1867)	8.5% (3373)	40.7% (16186)	23.4% (9287)	6.4% (2529)	11.9% (4730)
**FEMALES**									
**Estonia**	2007	1.3% (279)	1.0% (200)	4.1% (849)	6.2% (1288)	47.3% (9825)	23.3% (4834)	2.9% (612)	13.9% (2876)
	2018	0.6% (87)	1.4% (209)	5.0% (721)	3.6% (523)	42.0% (6065)	31.3% (4520)	4.4% (632)	11.7% (1684)
**Latvia**	2007	0.5% (132)	1.1% (276)	3.1% (801)	6.3% (1650)	49.5% (12906)	20.0% (5205)	3.1% (802)	16.5% (4288)
	2018	1.9% (365)	0.6% (114)	3.8% (719)	4.6% (877)	45.8% (8764)	26.4% (5051)	4.3% (828)	12.7% (2434)
**Lithuania**	2007	0.3% (63)	1.6% (376)	6.6% (1562)	7.7% (1837)	48.3% (11503)	20.6% (4919)	3.9% (929)	11.1% (2649)
	2018	0.7% (104)	1.5% (291)	3.9% (661)	4.0% (873)	44.1% (7993)	27.2% (4541)	4.6% (755)	13.9% (2306)

The total number of ASYR is in the brackets. *CVD* cardiovascular disease

ASYR from suicide was decreasing in all three countries for both sexes during the period 2007-2018 (Fig. 2c and d). Lithuanian males were still experiencing the heaviest suicide burden (1649) when compared to Estonian (950) and Latvian (1172) males in 2018. Male ASYR from suicide has decreased from 2502 (4.3%) in the year 2007 to 1649 (4.1%) in the year 2018 in Lithuania (see Table 1; for additional details on the calculation of age-standardized years of life lost, see Additional file 1). The major burden of suicide mortality was concentrated among the working-age population (20-59 years) during the reference period (Fig. 3c and d).

Patterns of ASYR from substance use disorders again varied in all three Baltic countries (Fig. 2e and f). In Estonia, ASYR was fluctuating at the beginning and then started decreasing for both sexes. In Latvia, ASYR related to alcohol and drug use increased for males but decreased for females. In Lithuania, ASYR related to substance use decreased more than twice for both sexes.

ASYR from external causes of death were decreasing in the Baltic States for both sexes during the entire study period 2007-2018 (Fig. 2g and h). Furthermore, this progress showed quite a similar pattern across all three countries. In 2018, the burden of external causes mortality for males remained much lower (2075) in Estonia than in other two countries (3510 for Latvia and 3373 for Lithuania). Notably, this burden was about four times lower among females in each country.

## Discussion

In this study, we explored the burden of mental health-related mortality in Estonia, Latvia, and Lithuania from 2007 to 2018. The aim was to examine the changing pattern of mortality from mental disorders, suicides, alcohol and drug use disorders, and external causes of death. Better understanding what groups of causes of death related to mental health are the most responsible for years of life lost in population is particularly important for the post-soviet countries, where lacking comprehensive scientific evidence coincides with inadequate and/or poorly implemented policies. Such long-lasting contradiction persists despite a striking burden related to excess deaths such as suicides. Although the situation has been improving especially in reducing suicide mortality, the post-soviet region including the Baltic countries, Russia, and Belarus remains a hotspot of suicide mortality in Europe and globally [8].

Results of our study show that the proportion of ASYR from mental disorders in all three countries was relatively small when compared to other causes of death. However, it was much higher when including suicide, substance use-related causes of death, and external causes of death. Although it is obvious that not all external causes of death are related to mental health, we argue that there is a solid evidence showing a strong association between mental disorders and increased risk of dying from specific external causes such as suicide [12, 26].

We found two clear trends of mortality from mental disorders that are worth discussing. First, it was a clear increase in ASYR from mental disorders both for males and females in Latvia. This trend goes in line with mortality from mental disorder trends in other developed countries like Sweden, Germany or United Kindom [8]. However, there was no such trend in Estonia or Lithuania. On the contrary, Estonia showed a sudden decrease in ASYR from mental disorders starting from 2008, which was caused by reduced mortality from mental disorders due to use of alcohol.

Data from international mortality databases [8, 31, 32] show that mortality indicators from mental disorders (F00-F99) in the Baltic States are still relatively low when compared to other EU countries. For example, standardized mortality rate for mental and behavioural disorders in Lithuania in 2015 was 3 deaths per 100,000 population. While the same indicator in France was 16, Germany – 24, Netherlands – 36 deaths per 100,000 population [8]. Data from France show that majority of causes of death in mental and behavioural disorders group was dementias (F01, F03) which mainly concentrated among 65+ years age group. Such a pattern can be explained by the different overall causes of death structure in Western Europe and post-soviet countries. The Baltic States suffered a long-lasting health crisis which disturbed entering into the cardiovascular revolution that allowed Western Europe to maintain rapid progress in life expectancy [33]. As a result, cardiovascular diseases are still dominant cause of death, especially among elder population in Estonia, Latvia, and Lithuania. Nevertheless, standardized mortality rates for other mental health-related causes of death such as suicide and alcohol-related mortality remains very high in the Baltic States when compared to othe EU countries [8, 32].

Our findings indicate that during 2007-2018, the measurements of the burden of mental health-related mortality showed pronounced differences in patterns and directions of changes across the three seemingly similar countries still suffering from common problems from the soviet past. The trajectories of changes were also diverging for various mental health-related causes of death. ASYR from suicide and external causes of death were decreasing but remained very high in the international context. The proportion of ASYR from suicide when compared to ASYR from all deaths stagnated (in Lithuania) or even increased (in Estonia and Latvia). Lithuania was the only country which showed substantial decrease in ASYR from alcohol and drug use disorders.

Our study suggests about large ASYR disparities in mental health-related causes of death by sex and age group. The burden of mental health-related mortality was concentrated among males, especially of working ages. Findings confirm prior evidence showing large sociodemographic disparities in suicide in three countries [13, 34, 35]. A previous study on determinants of male suicide in Lithuania confirmed that lower educated, unemployed or economically inactive, non-married, residing or being born in rural areas experience a particularly high risk of suicide [13]. The same study also showed that residing in socioeconomically disadvantaged areas has an additional positive effect on increasing male suicide risk which persists even after controlling for major individual-level characteristics. These risk groups and persisting inequalities should be taken into account in planning and implementing mental health policies.

We found a substantial decrease in ASYR from alcohol and drug use-related causes of death for both males and females in Lithuania. It is notable that the observed decrease in this group of causes of death was mainly driven by a notable reduction in alcohol-related mortality. It is a noteworthy that the progress in reducing alcohol-related mortality from 2007 was an important contributor to increase in life expectancy [22]. This improvement was associated with important alcohol control policy measures implemented in 2007-2018 [22, 36]. During the reference period, Lithuania introduced several comprehensive alcohol control measures: banned alcohol advertising, shortened retail hours, raised excise tax, introduced criminal charges for drunk driving, reduced the blood alcohol concentration permitted for novice drivers, banned alcohol sales in petrol stations, increased legal age of drinking to 20 years [22, 37]. This evidence indicates a strong potential of further reduction of the mortality burden of mental health-related causes of death by reducing the harm of alcohol [38].

Current literature stresses the threat of growing importance of drug-related epidemics leading to notable mortality increases in some countries such as the US and Canada [39–41]. Although, with an exception of the UK, there is no significant evidence about similar scale crisis in Europe, there are some warning signs, including transient peaks in opioid-related consumption, poisoning, and mortality in the three Baltic countries [42]. We have included accidental poisoning by drugs (X42) which includes opioids, cocaine, etc. in our analysis. However, number of deaths due to alcohol poisoning was higher when compared to drug poisoning.

Mental health-related mortality had significant impact to overall mortality in the Baltic states. The total number of ASYR from suicide and substance use disorders decreased in all three countries for both males and females. However, in Estonia and Latvia, the share of the burden of these deaths in the total ASYR remained high and even increased. Thus, we suggest that improvement in suicide and substance use-related mortality lagged behind the improvement in overall mortality in Estonia and Latvia. However, that was not the case in Lithuania, which showed a significant decrease in substance use-related mortality. Both the total number of ASYR and share in overall mortality significantly decreased during the reference period.

Our study has several limitations which have to be mentioned. First of all, there are a few important methodological limitations related to the list of causes of death we used. It should be noted that results of previous studies and meta-analyses have shown that alcohol and illicit drug use is related to many causes of death and its groups [43, 44]. However, in this study, we have used a short list of the most numbered substance use-related causes of death such as alcohol or drug poisoning, alcohol liver disease, and others. Therefore, the total burden of substance use-related mortality could be underestimated in our study.

We also included external causes of death in our analyses dedicated to examining the burden of mental illness and substance use-related mortality. On one hand, it is very difficult to estimate the exact fraction of external causes of death related to alcohol use or mental health problems. Moreover, it is often difficult to determine causal relationships between specific mental disorders and specific external causes of death. On the other hand, there are numerous studies showing the relationship between substance use and external causes of death [24, 43, 44]. For example, the Lithuanian autopsy data show, that 56.8% of blood samples taken from the persons who died from external causes of death contained alcohol in blood [23]. In Estonia, alcohol in blood was found in 48.7% of males who died from suicide in 1981–1992 [24]. The most recent data on persons who died from external causes of death in Lithuania [45] shows that the share of positive blood samples remains very high (54.7%).

Mental health-related disorders such as substance use-related illnesses may lead to death. On the contrary, many health conditions increase the risk for a mental disorder or lengthen episodes of mental illness [4]. Therefore, the burden of mental health-related mortality is often underestimated by including only mental disorders and substance use-related mortality [1, 4]. Previous studies show that a large proportion of suicides are related to mental disorders [46, 47] and alcohol abuse [45]. Moreover, meta-analyses and population-based studies reported that mental disorders are independently associated with a substantial excess in all-cause mortality risk [4].

Recent two decades have been marked by important changes in the field of mental health globally. On one hand, there was increasing agreement that mental health and mental healthcare are obvious priorities for health policies [48]. On the other hand, there was increasing evidence that investments in the prevailing biomedical model do not result in expected outcomes. Important documents by World Health Organization [49], UN Human Rights Council [50], and other international, regional organizations have urged goverments and other stakebolders to move to such mental health policies and services that are fully in line with human rights-based approach and evidence.

The region of the Baltic States has seen a respectable development during the two most recent decades. However, further progress is needed in the areas of human rights and community services. Historically, mental health systems in the region have been affected for many decades by the legacy of institutionalization, social exclusion, over-medicalization, and discrimination. Thirty years of democracy and independence have not fully abandoned the effects of that legacy. Political will is needed to invest more not just in existing infrastructure and technology, but to change the system so that mental health policies and services are liberated from a legacy of discrimination.

## Conclusions

Our findings indicate that the burden of mental health-related mortality remained high and showed divergent temporal changes across the three countries. Major burden of mental health-related mortality was concentrated among workin-age males in Estonia, Latvia, and Lithuania. Furthermore, alcohol is likely to be an important determinant behind some mental health-related causes of death in the Baltic countries. In the context of the Baltic States and other post-soviet countries, fractions of various external causes of death and alcohol-related causes of death should be considered in assessing the total burden of mental health-related mortality.

## Supplementary Information

**Additional file 1.** Age-standardized years of life lost from major causes of death groups and specific mental health-related causes of death groups, in Estonia, Latvia, and Lithuania in 2007-2018.

## Data Availability

The datasets analysed during the current study are available from the corresponding author on reasonable request.

## Abbreviation

ASYR: Age-standardized years of life lost
