# Supplementary material for: The burden of mental health-related mortality in the Baltic States in 2007-2018
Source: BMC Public Health. 2022 Sep 19;22:1776. doi: 10.1186/s12889-022-14175-9 (PMC9487037; doi:10.1186/s12889-022-14175-9)
Supplement: Supplementary file 1 — Additional file 1. Age-standardized years of life lost from major causes of death groups and specific mental health-related causes of death groups, in Estonia, Latvia, and Lithuania in 2007-2018. [file 12889_2022_14175_MOESM1_ESM.docx]

**Additional file 1**

Age-standardized years of life lost from major causes of death groups and specific mental health-related causes of death groups, in Estonia, Latvia, and Lithuania 2007-2018

**Estonia, males**

| Year | Mental disorders | Suicide | Substance use | External causes | CVD | Cancer | Infections and resp. | All other | All causes |
| --- | --- | --- | --- | --- | --- | --- | --- | --- | --- |
| 2007 | 1197 | 1391 | 2805 | 5960 | 20893 | 10210 | 2500 | 4924 | **49880** |
| 2008 | 799 | 1302 | 2595 | 4778 | 20040 | 9839 | 2357 | 3926 | **45637** |
| 2009 | 538 | 1474 | 3071 | 3880 | 17944 | 9642 | 2251 | 3775 | **42574** |
| 2010 | 535 | 1219 | 2654 | 3606 | 17214 | 9733 | 1926 | 3271 | **40158** |
| 2011 | 491 | 1163 | 2925 | 3429 | 15538 | 9454 | 1978 | 3347 | **38326** |
| 2012 | 539 | 1260 | 3275 | 3089 | 15470 | 9488 | 2081 | 3194 | **38396** |
| 2013 | 399 | 1115 | 2298 | 2736 | 14615 | 9220 | 2126 | 3156 | **35664** |
| 2014 | 402 | 1315 | 2789 | 2572 | 14132 | 9405 | 2116 | 3139 | **35870** |
| 2015 | 386 | 1098 | 2278 | 2117 | 13359 | 9368 | 1876 | 3403 | **33885** |
| 2016 | 354 | 933 | 2335 | 2165 | 13209 | 8750 | 1891 | 3938 | **33575** |
| 2017 | 411 | 1117 | 2726 | 1843 | 12469 | 8831 | 1730 | 3312 | **32439** |
| 2018 | 371 | 950 | 2329 | 2075 | 12036 | 9070 | 1922 | 3186 | **31940** |

**Estonia, females**

| Year | Mental disorders | Suicide | Substance use | External causes | CVD | Cancer | Infections and resp. | All other | All causes |
| --- | --- | --- | --- | --- | --- | --- | --- | --- | --- |
| 2007 | 279 | 200 | 849 | 1288 | 9825 | 4834 | 612 | 2876 | **20764** |
| 2008 | 226 | 280 | 842 | 1014 | 9541 | 4788 | 599 | 2287 | **19576** |
| 2009 | 200 | 215 | 731 | 972 | 8903 | 4860 | 568 | 1906 | **18356** |
| 2010 | 132 | 195 | 919 | 790 | 8583 | 4527 | 538 | 1865 | **17550** |
| 2011 | 137 | 208 | 749 | 757 | 7706 | 4784 | 596 | 1743 | **16680** |
| 2012 | 133 | 190 | 827 | 749 | 7478 | 4718 | 477 | 1833 | **16405** |
| 2013 | 91 | 197 | 651 | 728 | 7593 | 4568 | 570 | 1779 | **16178** |
| 2014 | 89 | 243 | 685 | 553 | 7084 | 4766 | 675 | 1678 | **15774** |
| 2015 | 86 | 184 | 715 | 700 | 6820 | 4618 | 509 | 1816 | **15448** |
| 2016 | 93 | 190 | 735 | 618 | 6499 | 4645 | 610 | 1893 | **15282** |
| 2017 | 117 | 278 | 665 | 390 | 6231 | 4542 | 609 | 1707 | **14539** |
| 2018 | 87 | 209 | 721 | 523 | 6065 | 4520 | 632 | 1684 | **14442** |

**Latvia, males**

| Year | Mental disorders | Suicide | Substance use | External causes | CVD | Cancer | Infections and resp. | All other | All causes |
| --- | --- | --- | --- | --- | --- | --- | --- | --- | --- |
| 2007 | 410 | 1503 | 1969 | 6860 | 26871 | 10521 | 3081 | 6486 | **57701** |
| 2008 | 420 | 1800 | 2392 | 5588 | 23782 | 10724 | 2575 | 6425 | **53704** |
| 2009 | 438 | 1788 | 2543 | 4623 | 22386 | 10413 | 2394 | 5574 | **50158** |
| 2010 | 502 | 1647 | 2575 | 4945 | 22193 | 10522 | 2284 | 4970 | **49639** |
| 2011 | 665 | 1589 | 2245 | 4191 | 20895 | 10261 | 2440 | 4592 | **46877** |
| 2012 | 546 | 1663 | 2446 | 4124 | 20303 | 10283 | 2374 | 4405 | **46144** |
| 2013 | 623 | 1430 | 2194 | 3922 | 19599 | 10133 | 2721 | 4004 | **44624** |
| 2014 | 527 | 1429 | 2219 | 4497 | 19185 | 10173 | 2355 | 3950 | **44337** |
| 2015 | 748 | 1373 | 2541 | 3943 | 18274 | 9493 | 2366 | 3708 | **42445** |
| 2016 | 667 | 1328 | 2329 | 3713 | 18298 | 9726 | 2435 | 3702 | **42198** |
| 2017 | 721 | 1294 | 2175 | 3543 | 18414 | 9896 | 2426 | 3843 | **42312** |
| 2018 | 802 | 1172 | 2246 | 3510 | 17758 | 9551 | 2543 | 4185 | **41766** |

**Latvia, females**

| Years | Mental disorders | Suicide | Substance use | External causes | CVD | Cancer | Infections and resp. | All other | All causes |
| --- | --- | --- | --- | --- | --- | --- | --- | --- | --- |
| 2007 | 132 | 276 | 801 | 1650 | 12906 | 5205 | 802 | 4288 | **26061** |
| 2008 | 160 | 291 | 845 | 1376 | 11315 | 5057 | 762 | 3823 | **23629** |
| 2009 | 157 | 297 | 803 | 1118 | 10717 | 5394 | 796 | 3731 | **23012** |
| 2010 | 144 | 178 | 744 | 1207 | 10610 | 5431 | 686 | 3558 | **22559** |
| 2011 | 191 | 222 | 715 | 1032 | 10048 | 5103 | 731 | 3149 | **21191** |
| 2012 | 221 | 220 | 697 | 1093 | 10092 | 5019 | 708 | 2783 | **20833** |
| 2013 | 208 | 203 | 777 | 1125 | 9995 | 4989 | 1032 | 2474 | **20803** |
| 2014 | 200 | 244 | 719 | 1001 | 9555 | 4976 | 733 | 2083 | **19511** |
| 2015 | 255 | 238 | 678 | 876 | 9380 | 5191 | 672 | 2210 | **19499** |
| 2016 | 230 | 238 | 712 | 886 | 9114 | 4850 | 765 | 2464 | **19259** |
| 2017 | 262 | 199 | 606 | 988 | 8888 | 4978 | 788 | 2440 | **19150** |
| 2018 | 365 | 114 | 719 | 877 | 8764 | 5051 | 828 | 2434 | **19152** |

**Lithuania, males**

| Year | Mental disorders | Suicide | Substance use | External causes | CVD | Cancer | Infections and resp. | All other | All causes |
| --- | --- | --- | --- | --- | --- | --- | --- | --- | --- |
| 2007 | 172 | 2502 | 4680 | 8158 | 23136 | 10646 | 4148 | 5296 | **58739** |
| 2008 | 181 | 2731 | 4150 | 6856 | 21552 | 10117 | 3636 | 4702 | **53926** |
| 2009 | 134 | 2804 | 3075 | 5820 | 20658 | 10156 | 3166 | 4447 | **50260** |
| 2010 | 93 | 2494 | 3027 | 5878 | 20443 | 10155 | 2728 | 4472 | **49290** |
| 2011 | 102 | 2499 | 2807 | 5053 | 20029 | 9912 | 2874 | 4363 | **47637** |
| 2012 | 119 | 2327 | 2805 | 5178 | 19450 | 9662 | 2853 | 4171 | **46565** |
| 2013 | 77 | 2761 | 2722 | 4790 | 19106 | 9393 | 3086 | 4269 | **46204** |
| 2014 | 112 | 2391 | 2566 | 4553 | 17976 | 9700 | 2703 | 4262 | **44263** |
| 2015 | 110 | 2246 | 2576 | 4021 | 18377 | 9889 | 2876 | 4490 | **44585** |
| 2016 | 120 | 2120 | 2506 | 4061 | 18061 | 9642 | 2857 | 4466 | **43832** |
| 2017 | 166 | 1828 | 2180 | 3508 | 16875 | 9173 | 2509 | 4205 | **40443** |
| 2018 | 130 | 1649 | 1867 | 3373 | 16186 | 9287 | 2529 | 4730 | **39752** |

**Lithuania, females**

| Years | Mental disorders | Suicide | Substance use | External causes | CVD | Cancer | Infections and resp. | All other | All causes |
| --- | --- | --- | --- | --- | --- | --- | --- | --- | --- |
| 2007 | 63 | 376 | 1562 | 1837 | 11503 | 4919 | 929 | 2649 | **23838** |
| 2008 | 47 | 421 | 1354 | 1683 | 10927 | 5290 | 937 | 2485 | **23145** |
| 2009 | 34 | 411 | 971 | 1270 | 10550 | 4902 | 842 | 2188 | **21168** |
| 2010 | 54 | 419 | 875 | 1330 | 10366 | 4754 | 675 | 2273 | **20746** |
| 2011 | 56 | 445 | 941 | 1189 | 9793 | 4928 | 670 | 2090 | **20113** |
| 2012 | 64 | 380 | 841 | 1250 | 9548 | 4687 | 762 | 1997 | **19528** |
| 2013 | 52 | 485 | 943 | 1076 | 9387 | 4653 | 869 | 2142 | **19608** |
| 2014 | 54 | 347 | 841 | 1039 | 8922 | 4539 | 665 | 2245 | **18654** |
| 2015 | 52 | 395 | 817 | 1005 | 9103 | 4817 | 887 | 2116 | **19193** |
| 2016 | 64 | 284 | 780 | 918 | 8727 | 4746 | 890 | 2148 | **18557** |
| 2017 | 121 | 344 | 671 | 912 | 8278 | 4621 | 729 | 2280 | **17956** |
| 2018 | 104 | 291 | 661 | 873 | 7993 | 4541 | 755 | 2306 | **17524** |

**Note.** Standard life expectancy for the year 2050 provided by WHO Global Health Estimates and the Standard European population of 2013 was applied to calculations.
